# 肺癌外科手术切口的演变与发展趋势

**DOI:** 10.3779/j.issn.1009-3419.2016.06.08

**Published:** 2016-06-20

**Authors:** 冬 谢, 昶 陈, 格宁 姜

**Affiliations:** 200433 上海，同济大学附属上海市肺科医院胸外科 Department of Thoracic Surgery, Shanghai Pulmonary Hospital, Tongji University, Shanghai 200433, China

**Keywords:** 肺肿瘤, 手术切口, 胸腔镜, 单孔胸腔镜, Lung neoplasms, Surgical incisions, Video-assisted thoracic surgery, Single-port VATS

## Abstract

微创、安全以及无瘤原则，是影响肺癌外科手术切口选择的最重要因素，从现有经验来看，把握手术指征，选择恰当的切口，掌握微创手术技巧，各种微创全胸腔镜手术均是安全可靠的切口入路，但如果术中无法满足安全或无瘤原则，应果断更改为开胸手术；开胸手术入路仍是肺癌外科治疗的基石，特别是应用于中央型病灶以及复杂肺癌外科手术。单孔VATS技术与3D胸腔镜、经自然孔道内镜技术、虚拟现实可视化技术以及机器人VATS技术的结合，是肺癌外科手术切口未来发展的方向。

## Abstract (en)

Minimally invasive, safe and tumor-free are the main principles of the choice of surgical incision in lung cancer surgery. In recent years, with the advances in minimally invasive techniques, single-port video assisted thoracic surgery (VATS), robot-assisted thoracoscopic (RATS), suboxiphoid single-port VATS, simultaneous bilateral VATS pulmonary resection, are emerging approaches, single-port VATS has become one of the most exciting new developments in minimally invasive thoracic surgery in recent years. This paper reviews the evolution and trends of surgical incision in lung cancer surgery.

## 1 手术切口的演变与历史沿革

1933年，第一例肺癌外科手术为Graham的左全肺切除。肺癌手术切口的发展分为以下三个阶段：①经典的开胸切口，主要是后外侧切口，肋床入路或肋间入路，需切开背阔肌及前锯肌；②保留胸壁肌肉的muscle-sparing（MS）切口，根据肿瘤部位和美容学要求，可选择腋下竖切口、胸前外侧切口、保留前锯肌的后外侧切口等，切口长度约8 cm-12 cm。③胸腔镜为代表的微创外科时代，包括经典3-4孔VATS、单操作孔VATS、单孔VATS以及机器人辅助VATS等。

肺癌手术切口的演变与以下因素相关：①肺癌疾病谱的改变以及影像学的早期发现，肺癌由最初中央型、巨块型病灶为主，转变为周围型结节、小结节或磨玻璃结节为主；②胸腔镜等手术器械的发展与进步；③微创理念以及微创手术技巧的积累。

随着肺癌部位的不同，还先后涌现了前外侧切口、大S形切口、正中劈开切口、L型切口、横断胸骨的蛤式切口等，近年来，随着微创技术的进步，逐步出现了单操作孔电视辅助胸腔镜手术（video-assisted thoracic surgery, VATS）、单孔VATS、机器人辅助胸腔镜切除术（robotic-assisted thoracoscopic resection, RATS）、剑突下单孔VATS、双侧同期VATS肺切除术、跨纵隔单侧进胸双侧肺切除术等新兴入路，特别是单孔VATS，目前在国内外开展已呈燎原之势。本文拟对近年来，肺癌外科手术切口的发展趋势予以总结。

## 2 单孔VATS肺癌外科手术

### 2.1 单孔VATS的起源与发展

尽管目前并无标准的定义，单孔VATS肺叶切除手术概念包括：使用单孔切口（3 cm-5 cm），软性胸撑撑开主操作孔；完全腔镜下实施的解剖性肺叶切除和系统肺门、纵隔淋巴结清扫术。

1998年，单孔VATS最早由Migliore等^[1, 2]^率先开展，2004年，Rocco等^[3]^明确提出单孔VATS的概念，并于2013年，报道了600余例以肺楔形切除、活检等简单操作为主的单孔VATS手术^[4]^；2011年，Gonzalez-Rivas等^[5-7]^报道了首例单孔全腔镜肺叶切除术，2013年报道了首个解剖性肺切除的102例报道^[8]^。此后，Gonzalez-Rivas团队又率先开展了单孔VATS下全肺切除^[9, 10]^、肺动脉袖式切除^[11]^、支气管袖式切除^[12]^、解剖性肺段切除^[13]^、气管及隆凸切除术^[14]^等。中国大陆地区，同济大学上海市肺科医院朱余明等在国内率先大规模开展单孔VATS手术治疗早期肺癌^[14-21]^，2012年5月首次开展单孔VATS肺叶切除术，2014年在全国年会首次报道单中心1, 063例单孔VATS手术，截至2015年底，该中心单孔VATS手术总量超过4, 000例。

### 2.2 体位、站位以及切口的选择

患者取侧卧位，手术的切口位于腋前线至腋中线水平，第4或第5肋间，切口长度3 cm-4 cm；选择合适的切口位置对于解剖肺门管性结构和纵隔淋巴结清扫至关重要，如切口位置靠腋中线，则靠近肺门，易于解剖，便于清扫第7组淋巴结，但一次性切割缝合器置入角度不理想；如切口位置靠前，则远离肺门，清扫隆凸下淋巴结较困难，但一次性切割缝合器置入角度较理想。术者站在患者前面操作，助手站在患者前面或后面，术中腔镜位置并不固定，常位于切口的两端，以皮肤切端作为支点。不撑开肋间，采用软性切口保护套撑开肌肉，完全在胸腔镜视野下，使用腔镜器械进行解剖性切除。

### 2.3 单孔VATS操作经验

单孔VATS术中放射状牵引肺，改拉为推；根据手术步骤摇床以增加肺的暴露。全肺或下叶切除可采用从下向上逐步推进的方式；上叶切除，可采用从下向上逐步推进方式；无肺裂者，可最后处理肺裂；处理中叶静脉或舌段静脉时，如采用endo-GIA处理角度困难，可采用血管钛夹处理。

术中器械交叉，肺门血管和支气管充分游离，尽量鞘膜内处理，骨骼化游离，有利于切割缝合器的置入。采用内镜器械分离肺门血管主干，对于肺动脉、肺静脉主干均用内镜下切割缝合器缝合切断，对于直径小于3 mm的肺动脉分支可采用内镜切割缝合器，或用内镜打结、钛夹钳钳夹、hemolok钳夹结合超声刀或结扎速处理。

### 2.4 单孔VATS的优势

目前单孔腔镜下能够完成绝大多数解剖性肺叶切除、肺段切除、双叶切除；全肺切除，支气管、血管的袖式肺切除，隆凸及气管手术仅见于少量报道，与传统三切口相比，单孔VATS切口减少，主要优势在于仅损失一个肋间神经，有望进一步降低术后切口疼痛，缓解胸壁麻木，还原开胸下的视角，独特的视野，尤其对上纵隔的显示具有明显的优势，手术过程符合肿瘤原则。

### 2.5 单孔VATS手术安全性与早期结果

绝大多数单孔VATS的术中风险来自于术中出血^[22]^，Gonzalez-Rivas等^[18]^探讨了单孔VATS术中出血的控制，应首先采用压迫止血，评估出血程度，如出血程度较轻，或为较小的肺动脉分支出血，可考虑在单孔镜下缝合或钛夹钳夹止血；如果单孔下难以操作，可首先考虑中转2孔或3孔VATS；如出血情况较严重，应迅速中转开胸。随着经验的积累，单孔手术能够达到传统VATS肺癌系统淋巴结清扫的要求，单孔也并不会必然增加围手术期死亡率和并发症率^[23]^。谢冬等^[15]^报道了1, 063例单孔VATS手术，为目前文献报道的，单中心最大报道，充分证实了单孔胸腔镜手术的安全性和可行性。

## 3 RATS

RATS优势在于：三维视野下定位精确，手术姿态稳定；符合人体工学的手术器械，器械再定位容易，过滤手颤抖，移动度缩小，可完成精确的缝合，结扎；其缺点包括：术中缺乏大局观和触觉感，费用高，设备安装耗时等。目前已开展RATS肺叶切除术、RATS解剖性肺段切除以及RATS支气管袖式切除等，Augustin等^[24]^报告了75例RATS肺叶切除的临床经验，其切口设计和操作流程与胸腔镜相似，其安全性和可靠性类似于VATS肺叶切除术。多中心研究显示，RATS肺叶切除能够安全有效的应用于早期NSCLC，长期生存率与开胸或常规VATS一致^[25]^。由于操作的稳定性，RATS特别适用于复杂肺癌外科手术的操作。

## 4 剑突下单孔VATS手术

2014年8月，台湾Liu等^[26]^率先开展经剑突下径路单孔VATS肺叶切除术。大陆地区，2014年9月，由上海市肺科医院胸外科赵德平、蒋雷等率先开展剑突下单孔VATS手术，目前，单孔剑突下VATS超过600例。蒋雷等还率先报道了剑突下单孔同期双侧肺切除术^[21]^。该手术径路的特点是患者取半侧卧位（45度）从剑突下建立单孔通路，可以进入左侧或右侧胸腔，单孔下完成肺切除的操作。该手术径路不损伤肋间神经，可有效避免肋间切口术后长期顽固性疼痛；此外，该切口对于双侧多原发肺癌同期手术具有显著优势，单孔下可以同期完成双侧肺切除术。缺点是潜行径路较长，普通的器械难以到达解剖结构，需要特殊的加长器械；左侧手术受心脏的阻隔，视野较差，操作不便，右侧操作较顺利；隆凸下纵隔淋巴结的清扫较为困难；上叶切除术中出血难以控制。

## 5 胸腔镜手术应用范围扩大化

VATS肺叶切除用于早期NSCLC的治疗地位已得到确认，其近期临床效果优于开胸手术；其远期临床效果不差于甚至优于开胸手术。局部晚期NSCLC的VATS指征逐步扩大，部分中心开展了VATS袖式肺叶切除术^[27]^，VATS全肺切除，VATS肺癌合并胸壁切除^[28]^以及新辅助化疗后VATS肺切除术等。中央型肺癌，气道受侵犯，未侵犯周围重要血管，无需血管成形者，可尝试VATS支气管袖式切除或全肺切除。肿瘤侵犯局部胸壁，未侵透肋间肌，切除后胸壁缺损不大，无需行胸壁重建者，可试行VATS肺叶切除合并部分胸壁切除。腔镜下胸壁切除可采用特殊的肋骨切断器械。Hennon等^[29]^开展73例全胸腔镜局部晚期肺癌根治切除，其近期效果均与开胸相似；腔镜组和开胸组在总生存时间和无瘤生存时间上均无显著差异，但要求术者具有丰富的VATS手术经验。

## 6 其他手术入路

### 6.1 经自然腔道内镜外科学手术（natural orifice transluminal endoscopic surgery, NOTES）

NOTES体表无需切口，NOTES是利用人体自然开口和管腔，将内镜穿破管壁进入体腔，进行内镜下手术的外科学。多数研究应用粘膜下安全活瓣技术，经口腔食管径路进入胸腔操作：应用标准胃镜，首先清洁灌洗食管内膜；注射生理盐水至粘膜下，确认针尖进入粘膜下后，注入高压CO_2_气体，进入粘膜下层，使用球囊钝性向食管远端分离粘膜下层，形成10 cm长的粘膜下隧道，在隧道末端切开固有肌层，然后胃镜通过肌层切口进入胸腔进行手术操作。Moreira-Pinto等^[30]^采用猪模型，开展了经口腔食管径路肺叶切除（胸部单孔辅助）；Liu等^[31]^采用犬动物模型，证实经口腔气管径路肺活检可进行部分胸膜肺活检，与常规腔镜手术相比，具有类似的安全性和有效性。NOTES优势在于体表无切口，微创，但NOTES在肺癌外科治疗中的应用有其特殊风险：食管肌层切开位置需要避开心脏、大血管等重要结构；食管只有三层管壁结构，愈合能力较胃差，一旦发生瘘将产生严重的胸腔内感染；因此NOTES目前仍停留在动物实验阶段。

### 6.2 经颈部径路VATS手术

经颈部切口VATS手术思路起源于颈部纵隔镜入路，通过颈部单个操作孔，可以完成双侧肺手术。Assouad等^[32]^采用经颈部径路的实验研究，在10例尸体上经颈部，建立VATS径路，单孔下可行纵隔淋巴结及双侧胸膜的活检。Zielinski等^[33]^尝试开展了经颈部径路VATS肺叶切除术。Liberman等^[34]^报道：不伴有胸壁受累的病灶，可经颈部单孔，行VATS双侧肺胸膜活检，肺活检，胸膜固定术。经颈部径路手术的优势在于避免术后胸痛，可同期处理双侧肺部病变；左侧操作有主动脉弓的阻隔，特别适用于右肺病变的活检与切除；其缺点在于，术后颈部切口不够美观，术后引流管从颈部引出，引流方向与重力方向相反，引流效果较差，目前仍停留在动物实验阶段。

## 7 总结与展望

微创、安全以及无瘤原则，是影响肺癌外科手术切口选择的最重要因素，从现有经验来看，把握手术指征，选择恰当的切口，掌握微创手术技巧，各种微创全胸腔镜手术均是安全可靠的切口入路，但如果术中无法满足安全或无瘤原则，应果断更改为开胸手术；开胸手术入路仍是肺癌外科治疗的基石，特别是应用于中央型病灶以及复杂肺癌外科手术。单孔VATS技术与3D胸腔镜、经自然孔道内镜技术、虚拟现实可视化技术以及机器人VATS技术的结合，是肺癌外科手术切口未来发展的方向。
